# Neural precursor cells in the ischemic brain – integration, cellular crosstalk, and consequences for stroke recovery

**DOI:** 10.3389/fncel.2014.00291

**Published:** 2014-09-16

**Authors:** Dirk M. Hermann, Luca Peruzzotti-Jametti, Jana Schlechter, Joshua D. Bernstock, Thorsten R. Doeppner, Stefano Pluchino

**Affiliations:** ^1^Chair of Vascular Neurology, Dementia and Cognitive Health of the Elderly, Department of Neurology, University Hospital EssenEssen, Germany; ^2^John van Geest Centre for Brain Repair, Department of Clinical Neurosciences, NIHR Biomedical Research Centre, and Wellcome Trust-Medical Research Council Stem Cell Institute, University of CambridgeCambridge, UK

**Keywords:** cell therapy, neurogenesis, stroke, blood–brain barrier, brain plasticity, neuroprotection

## Abstract

After an ischemic stroke, neural precursor cells (NPCs) proliferate within major germinal niches of the brain. Endogenous NPCs subsequently migrate toward the ischemic lesion where they promote tissue remodeling and neural repair. Unfortunately, this restorative process is generally insufficient and thus unable to support a full recovery of lost neurological functions. Supported by solid experimental and preclinical data, the transplantation of exogenous NPCs has emerged as a potential tool for stroke treatment. Transplanted NPCs are thought to act mainly via trophic and immune modulatory effects, thereby complementing the restorative responses initially executed by the endogenous NPC population. Recent studies have attempted to elucidate how the therapeutic properties of transplanted NPCs vary depending on the route of transplantation. Systemic NPC delivery leads to potent immune modulatory actions, which prevent secondary neuronal degeneration, reduces glial scar formation, diminishes oxidative stress and stabilizes blood–brain barrier integrity. On the contrary, local stem cell delivery allows for the accumulation of large numbers of transplanted NPCs in the brain, thus achieving high levels of locally available tissue trophic factors, which may better induce a strong endogenous NPC proliferative response. Herein we describe the diverse capabilities of exogenous (systemically vs. locally transplanted) NPCs in enhancing the endogenous neurogenic response after stroke, and how the route of transplantation may affect migration, survival, bystander effects and integration of the cellular graft. It is the authors’ claim that understanding these aspects will be of pivotal importance in discerning how transplanted NPCs exert their therapeutic effects in stroke.

## INTRODUCTION

Ischemic stroke represents the most common cause of serious morbidity and the second most common cause of mortality in industrialized countries (Donnan et al., 2008). While a certain degree of spontaneous recovery of lost functions takes place in some stroke patients, the majority never regain full functional independence and ultimately suffer from a reduced quality of life (Lloyd-Jones et al., 2009). Clearly, this health burden represents a major unmet clinical need that may in part be fulfilled via a detailed understanding of the mechanisms driving neurological recovery after stroke.

In animal models, the mobilization and recruitment of NPCs from the major stem cell niches within the central nervous system [CNS; i.e., the sub-ventricular zone (SVZ) of the lateral ventricles and the sub-granular zone of the dentate gyrus (DG)] are essential compensatory responses after an ischemic insult (Arvidsson et al., 2002). However, while it is known that endogenous NPCs do positively enhance the brain’s own restorative potential via trophic influences on the ischemic microenvironment, the overall neurogenic response after stroke is insufficient for a number of reasons, which include the limited survival of NPCs, their transient mobilization from the neurogenic niches and their incomplete integration within damaged brain circuitries (Thored et al., 2007).

The observation that NPCs can be harvested from the adult brain and used therapeutically in animal models of stroke argues in favor of the potential utility of cell-based therapies in ischemic stroke. We have shown that the injection of somatic mouse NPCs ameliorates the clinicopathological features of stroke in relevant murine models by reducing secondary neurodegeneration, decreasing glial scar formation, promoting endogenous neurogenesis and stabilizing blood–brain barrier (BBB) integrity (Bacigaluppi et al., 2009; Doeppner et al., 2012). Grafted NPCs adapt to the ischemic microenvironment and facilitate homeostasis via the secretion of numerous tissue trophic factors that have beneficial effects on endogenous brain cells, as well as modulatory actions on both innate and adaptive immune responses (Pluchino and Cossetti, 2013). This concept has now come to be known as functional stem cell multipotency, and it is one of the core tenants behind the use of NPC grafts in attempt to boost the recovery potential of the ischemic brain (Martino and Pluchino, 2006).

However, there remains an enormous need to understand how the complex interactions of stem cell grafts with the ischemic brain may be affected by the route and timing of cell delivery. In particular, we still have to define how NPCs should best be administered in order to enhance endogenous restorative responses that depend on (i) the homing, survival and integration of transplanted NPCs, (ii) the proliferation of the host’s endogenous NPCs, (iii) the modification of the cerebral microenvironment, and (iv) the remodeling of ischemic tissue via actions that include the modification of glial responses and the promotion of neuronal plasticity.

Herein, we summarize current knowledge with regard to how transplanted NPCs interact with host tissues, aiming to identify how exogenously delivered NPCs may eventually be used to promote neurological recovery in mouse models of ischemic stroke.

## REGULATION OF ENDOGENOUS NEUROGENESIS AFTER STROKE

The SVZ is situated within the lateral walls of the lateral ventricles and is composed of four main cell types: ciliated ependymal cells (type E), slowly proliferating stem cells (type B), transient amplifying progenitors (type C) and proliferating neuroblasts (type A; Mirzadeh et al., 2008). After an ischemic stroke that involves the striatum, the number of type A and C cells in the SVZ is persistently increased, while type B and E cells undergo a period of transient proliferation (Zhang et al., 2004, 2007). Increases in mitotic activity within the SVZ appear to peak between 7 and 10 days, subsequently decrease during weeks 3–5 post-stroke, and thereafter continues at lower levels over the course of the following year (Arvidsson et al., 2002; Parent et al., 2002; Thored et al., 2006). This suggests that the SVZ may serve as a constant reservoir of new neurons after stroke even in the chronic phases of recovery, and thereby offers an extended window of opportunity for therapeutic intervention.

Signals that stimulate the stroke-induced neurogenic response have yet to be fully elucidated, but likely involve the interplay of morphogens, growth factors, and inflammatory mediators. Several groups have found that the notch pathway stimulates SVZ cell proliferation and neurogenesis after stroke (Androutsellis-Theotokis et al., 2006; Wang et al., 2009). Other signaling pathways that appear to be important for stroke-induced neurogenesis include retinoic acid (RA), sonic hedgehog (SHH), and bone morphogenic protein (BMP; Chou et al., 2006; Plane et al., 2008; Sims et al., 2009; Kernie and Parent, 2010). Soluble growth factors, such as basic fibroblast growth factor (bFGF), BDNF, epidermal growth factor (EGF), glial cell-derived neurotrophic factor (GDNF), erythropoietin (EPO), CNTF, transforming growth factor (TGF)-α, VEGF, and granulocyte-colony stimulating factor (G-CSF), have also been inextricably linked to stroke-induced neurogenesis (Kokaia et al., 1995; Planas et al., 1998; Kitagawa et al., 1999; Sun et al., 2003; Schneider et al., 2005; Tureyen et al., 2005; Leker et al., 2009; Kang et al., 2012). Inflammatory mediators have been shown to have variable effects on NPC proliferation, migration, survival, and incorporation within injured CNS circuitries (Peruzzotti-Jametti et al., 2014). Some studies have indeed reported that activated microglial cells can reduce NPC viability through the secretion of soluble molecules such as IFN-γ, IL-1β, IL-6, and tumour necrosis factor (TNF)-α or by direct cell-to-cell contact (Ben-Hur et al., 2003; Cacci et al., 2008). Other studies suggest instead that microglial cells can increase neurogenesis via TNF-α/ TNF-R2 interaction or insulin-like growth factor (IGF)-1 secretion (Heldmann et al., 2005; Thored et al., 2009). These differential effects of inflammation, which appear to either support or impair the adult neurogenic response, most likely depend on the phenotype of the inflammatory cells (and their cytokine production profile; Ekdahl et al., 2009).

**FIGURE 1:**
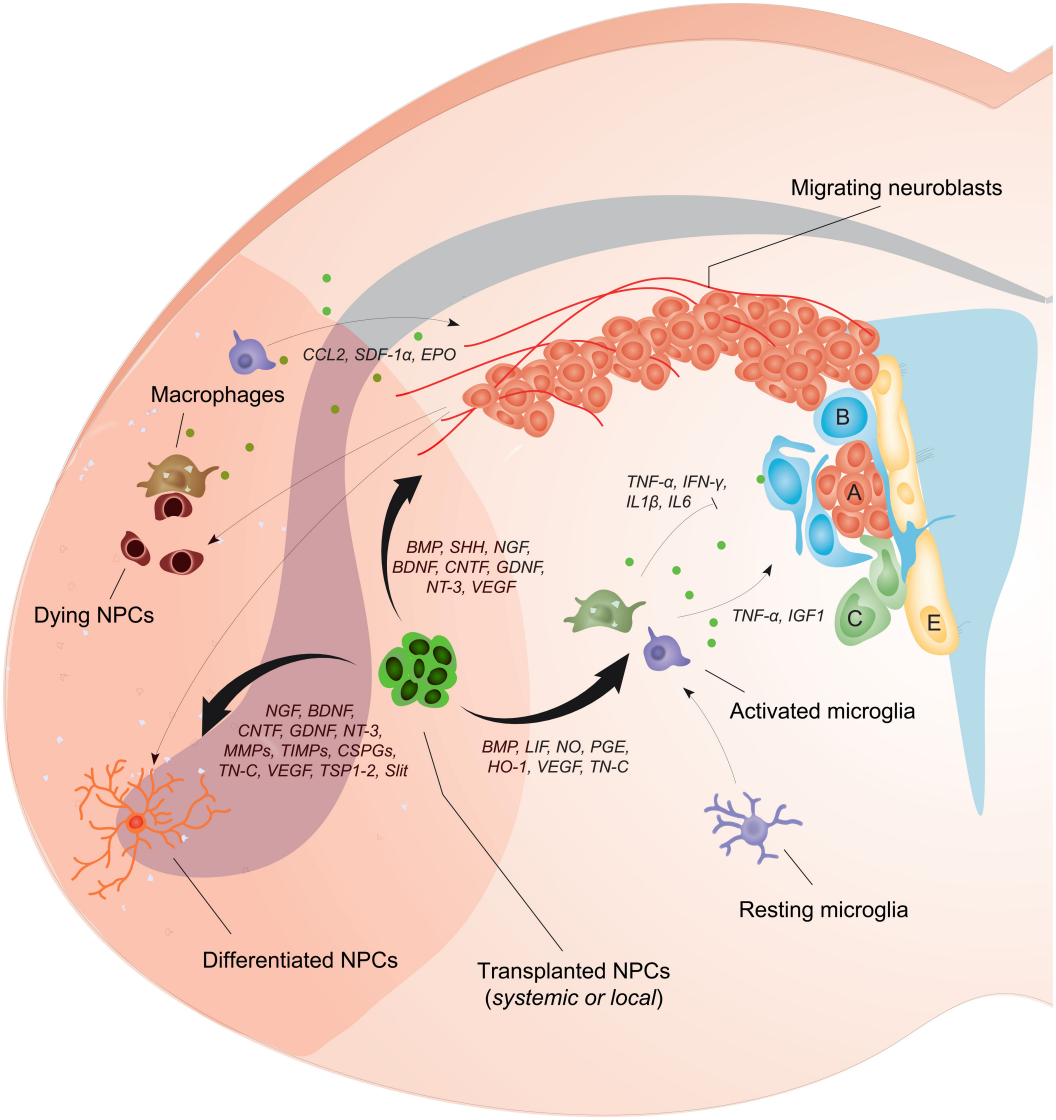
**Neural precursor cell (NPC) transplantation boosts the endogenous neurogenic response after stroke.** NPC proliferation within the SVZ is augmented after stroke leading to the generation of newly formed neuroblasts that migrate along vessels toward gradients of chemokines that are produced locally by glial and inflammatory cells (e.g., CCL2, SDF1-α, and EPO). The bystander effects of transplanted NPCs depend on the release of several factors (e.g., BMP, SHH, NGF, BDNF, CNTF, GDNF, NT-3, and VEGF) that can directly increase cell proliferation within the SVZ, potentiate neuroblasts migration, and augment peri-ischemic angiogenesis. Transplanted NPCs can also positively affect the differentiation of endogenous neuroblasts and plasticity within the ischemic tissue (via the secretion of NGF, BDNF, CNTF, GDNF, NT-3, MMPs, TIMPs, CSPGs, TN-C, VEGF, TSP1-2, Slit), or directly differentiate into post-mitotic neurons, astrocytes, or oligodendrocytes. Most importantly, transplanted NPCs secrete a plethora of soluble molecules that modulate the activation of host microglia/macrophages (e.g., BMP, LIF, NO, PGE, HO-1, VEGF, TN-C), thus modifying the release of inflammatory mediators that inhibit (e.g., TNF-α, IFN-γ, IL1β, IL6) or increase (e.g., TNF-α, IGF1) endogenous neurogenesis. Green dots represent mediators secreted by inflammatory cells.

Since NPCs can secrete many of the factors that regulate neurogenesis (Drago et al., 2013), and are also able to beneficially modulate inflammatory responses after CNS damage (Bacigaluppi et al., 2009; Cusimano et al., 2012), the possibility of exploiting NPC transplantation in an effort to augment endogenous neurogenesis and the brain’s spontaneous reparative processes (e.g., plasticity) after stroke is readily apparent. In this review, cellular and molecular interactions of NPCs with the brain environment have been illustrated in **Figure 1**. Effects of exogenously delivered NPCs in experimental models of stroke have been summarized in **Table 1**.

**Table 1:** Effects of exogenously delivered NPCs in experimental stroke models.

Study	Experimental paradigm	Observations
Andres et al. (2011a)	Intraarterial delivery of adult NPCs obtained from CCR2+/+ and CCR2-/- mice 24 h after transient (30 min) common carotid artery occlusion combined with 8% hypoxia in CCL2+/+ and CCL2-/- mice	Decreased homing of CCR2-/- NPCs compared with CCR2+/+ NPCs in CCL2+/+ mice. Decreased homing of CCR2+/+ NPCs in CCL2-/- compared with CCL2+/+ mice. Mice receiving CCR2+/+ NPCs showed significantly better neurological recovery than animals receiving CCR2-/- NPCs.
Andres et al. (2011b)	Intracerebral delivery of fetal human NPCs 7 days after permanent distal MCAO combined with transient (30 min) bilateral common carotid artery occlusion in rats	Increased dendritic plasticity in ipsi- and contralesional cortex after NPC delivery that coincided with functional neurological recovery. Increased corticocortical, corticostriatal, corticothalamic and corticospinal axonal sprouting from the contralesional hemisphere associated with transcallosal and corticospinal axonal sprouting. Reduced brain amyloid precursor protein accumulation.
Bacigaluppi et al. (2009)	Intravenous delivery of adult mouse NPCs 3 days after transient (45 min) proximal (intraluminal) MCAO in mice	Improved neurological recovery after NPC delivery. Small percentage of transplanted NPCs (< 1%) accumulated in the brain, integrating mainly in the infarct boundary zone, where most of the NPCs remained undifferentiated. Reduced secondary striatal and corpus callosum atrophy associated with downregulation of markers of inflammation, glial scar formation and neuronal apoptotic death.
Darsalia et al. (2011)	Intracerebral delivery of fetal human NPCs 48 h or 6 weeks after transient (30 min) proximal MCAO in rats	Better NPC survival after early than late NPC transplantation. Magnitude of NPC proliferation, migration, and neuronal differentiation was not influenced by transplantation time. Greater numbers of grafted NPCs did not result in greater numbers of surviving NPCs or increased neuronal differentiation.
Doeppner et al. (2012)	Intravenous or intracerebral delivery of adult mouse NPCs 6 h after transient (30 min) proximal (intraluminal) MCAO in mice	Intravenous and intracerebral NPC delivery similarly induced neurological recovery, but only intravenous NPC delivery yielded sustained neuroprotection that persisted in the post-acute stroke phase. Intracerebral NPF delivery associated with higher brain concentrations of BDNF, FGF, and VEGF. Intravenous, but not intracerebral NPC delivery stabilized blood–brain barrier, reduced activation of MMP9 and decreased formation of reactive oxygen species.
Hassani et al. (2012)	Intracerebral delivery of human conditionally immortalized neural stem cells CTX0E03 4 weeks after transient (60 min) proximal (intraluminal) MCAO in rats	Increased endogenous NPC proliferation in striatum of NPC treated rats. Significant proportion of proliferative cells expressed immature neuronal marker doublecortin. Increased proliferation of CD11b + microglial cells in NPC treated rats.
Jin et al. (2005)	Intravenous, intracerebral or intracerebroventricular delivery of embryonic mouse NPCs 24 h after permanent distal MCAO in rats	Brain entry of NPCs with accumulation in ischemic striatum and cortex observed using all three delivery strategies, intrastriatal transplants resulting in highest and intravenous transplants in lowest cell densities. Majority of cells expressing undifferentiated neuroepithelial (nestin) or neuronal (doublecortin) markers.
Mine et al. (2013)	Intracerebral delivery of fetal human NPCs 48 h after transient (60 min) proximal MCAO in T cell deficient rats	Subpopulation of NPCs exhibited differentiated neuronal phenotype at 6 and 14 weeks. Numbers of proliferating endogenous NPCs were elevated, and numbers of activated microglia/macrophages were reduced in ischemic striatum of NPC treated rats. Some grafted NPCs projected axons from striatum to globus pallidus. NPC treated rats showed improved neurological recovery.
Minnerup et al. (2011)	Intravenous delivery of embryonic mouse NPCs 24 h after photothrombotic stroke in immunosuppressed (cyclosporine A) rats	Improved neurological recovery associated with increased dendritic growth and branching, reduced endogenous neurogenesis and increased microglial activation in NPC treated rats.
Tang et al. (2014)	Intracerebral delivery of embryonic mouse NPCs 24 h after transient (120 min) proximal MCAO in young-adult (3 month-old) and aged (24 month-old) rats	Aged rats developed larger infarcts with worse neurological deficits than young-adult rats. Brain infarction and neurologic deficits were attenuated by NPC delivery in aged and young-adult rats. Number of surviving NPCs was similar in both age groups. Angiogenesis and neurogenesis were enhanced by NPCs in aged and young-adult rats.
Zhang et al. (2011)	Intracerebral delivery of fetal human NPCs 24 h after permanent distal MCAO in rats	Increased proliferation of endogenous NPCs in ipsilesional (ischemic) subventricular zone of rats receiving NPC grafts. Enhanced angiogenesis in peri-infarct cortex.

*List of major studies using ischemic stroke models, in alphabetic order.*

## EFFECTS OF TRANSPLANTED NPCs ON ENDOGENOUS NEUROGENESIS

In view of their intrinsic actions, the possibility of exploiting NPCs in an effort to augment *endogenous* neurogenesis has been the focus of intensive research efforts. Among the different routes of NPC delivery, there are several studies suggesting that the local (i.e., intracerebral or intracerebroventricular) administration of NPCs has the most relevant effects on endogenous neurogenesis. The local route of cell delivery indeed allows for a large number of cells to be administered, which facilitates the secretion of high concentrations of growth factors that ultimately promote the endogenous neurogenic response (Hao et al., 2014). In line with the need for an efficient accumulation of NPCs within the ischemic parenchyma, the intravenous administration of NPCs may be inferior with regard to the stimulation of neurogenesis. As such, when mouse embryonic stem (ES)-derived NPCs were transplanted 24 h after photothrombotic stroke in adult immunosuppressed rats, no effect on post-stroke neurogenesis of the SVZ was shown, and a decrease in newly generated neurons in the DG was observed (Minnerup et al., 2011).

The early transplantation of human NPCs has instead been proven highly effective in stimulating endogenous neurogenesis in rats when cells were delivered directly into the ischemic brain parenchyma. Human fetal NPCs, injected in the cortical peri-infarct tissue 24 h after permanent middle cerebral artery occlusion (MCAO) promoted cell proliferation in the SVZ (up to 15 days post-stroke) and increased angiogenesis in peri-infarct regions (Zhang et al., 2011). Intraparenchymal cell delivery has also yielded possible evidence of therapeutic potential when administered in the subacute and chronic phases of the stroke. A study with human fetal NPCs transplanted 2 days after MCAO showed that the intraparenchymal injection of NPCs was able to promote endogenous neurogenesis in multiple ways (Mine et al., 2013). Transplanted cells were able to induce a prolonged increase in Ki67^+^ proliferating cells within the SVZ, which was associated with an increase in the number of endogenous neuroblasts survival, migration and maturation in the striatum. This prolonged endogenous neurogenic response, which persisted up to 14 weeks, was accompanied by the long-term suppression of microglia/macrophage driven inflammatory responses (Mine et al., 2013). Similarly, when human conditionally immortalized NPCs (cell line CTX0E03) were transplanted in immunosuppressed rats 4 weeks after MCAO, the number of proliferating doublecortin (DCX)^+^ neuroblasts considerably increased in the ischemic striatum (Hassani et al., 2012). Interestingly, the authors attributed this finding to an increase (rather than a decrease) of proliferating microglia in the striatum (Hassani et al., 2012).

Despite these data, which highlight the promising effects of intraparenchymal NPC delivery, comparative studies of different routes and times of transplantation (without the use of confounding immunosuppressive regimens) must be performed in order to determine the optimal spatiotemporal settings that would allow for the ideal stimulation of endogenous neurogenesis in stroke. Additionally, given that the neurogenic potential of adult NPCs declines with age (Conover and Shook, 2011), the effect of NPC transplantation in aged brains also warrants investigation. Interestingly, a recent study has shown that the local NPC transplantation (24 h post-ischemia) is capable of similar increases in neurogenesis and angiogenesis in the ischemic striatum of both young and aged mice (Tang et al., 2014). The aforesaid, coupled with recent data showing increased neurogenesis in the SVZ of both young and aged animals following the local administration of human ES-derived NPCs, suggests that the ischemic environment may effectively be modified in order to restore deficient neurogenic responses in the aged brain as well (Jin et al., 2011).

## HOMING AND SURVIVAL OF TRANSPLANTED NPCs

In a comparative study evaluating intrastriatal, intracerebroventricular and intravenous NPC delivery (24 h after MCAO), the intrastriatal transplant of NPCs yielded the highest numbers of grafted cells within the ischemic brain (Jin et al., 2005). Intracerebroventricular transplantation into the lateral ventricle led to the survival of less cells, yet more cells were found when compared to intravenous NPC delivery (Jin et al., 2005). The reasons behind these observations are manifold in nature and may be linked to both the differential homing of grafted cells and their distinct survival profiles within the ischemic brain.

The mechanisms that regulate the homing of transplanted cells to the ischemic lesion are extremely similar to those that regulate the migration from within the endogenous NPC compartment (Jin et al., 2003; Zhang et al., 2009). Upon ischemia, EPO activates endothelial cells, which promote endogenous neuroblasts migration by secreting MMPs that degrade extracellular matrix (ECM) components (Wang et al., 2006). Migration of neuroblasts along the vessels is then modulated by the interaction of chemokine receptors on NPCs [e.g., C-X-C motif chemokine receptor (CXCR)-4 and C-C motif chemokine receptor (CCR2)] with molecules secreted by activated neuronal and glial cells within the ischemic lesion [e.g., stromal-derived factor (SDF)-1α and C-C chemokine ligand (CCL)-2, respectively; Robin et al., 2006; Yan et al., 2007].

When exogenous NPCs are administered intravenously, the crossing of the BBB involves a further degree of complexity. The CCL2/CCR2 interaction has been demonstrated to be critical for transendothelial recruitment of intraarterially delivered NPCs in response to ischemic injury (Andres et al., 2011a). Stem cells in circulation directly enter the injured brain through endothelial rolling and adhering on cadherins (VCAM-1) and integrins that become selectively up-regulated within the zone of ischemic damage (Mueller et al., 2006). This phenomenon, coupled with the disturbance of BBB integrity that occurs as a consequence of ischemia, is responsible for the *pathotropism* of transplanted NPCs for ischemic tissue that occurs after systemic delivery. Interestingly, intravenous transplantation of NPCs has the unique advantage of stabilizing the BBB, via mechanisms that involve a reduction of MMP9 expression and reactive oxygen species (ROS), as we recently showed after the transplantation of adult NPCs at 6 h after transient MCAO (Doeppner et al., 2012). Despite the fact that systemic NPC transplantation yields fewer cells in the ischemic brain parenchyma, current evidence suggests that intravenous injection is clearly able to promote neuronal/glial survival at delayed time-points (Bacigaluppi et al., 2009; Doeppner et al., 2012). As such, while intracerebrally transplanted adult mouse NPCs transiently improved motor coordination during the first 1–2 weeks after MCAO in mice, intravenous NPC transplantation resulted in persistent motor coordination improvements that persisted over at least 8 weeks post-stroke (Doeppner et al., 2012).

We have previously shown that upon the intravenous transplantation of adult mouse NPCs in the subacute stroke phase (72 h post-ischemia) only a small minority of transplanted cells (approximately 0.3% based on systematic counts) accumulate in the brain (Bacigaluppi et al., 2009). Notably, within the first 72 h post transplantation, intravenously injected NPCs were found both in the ischemic and contralesional non-ischemic hemisphere (Bacigaluppi et al., 2009). In the subsequent 7 days the NPCs in the contralesional hemisphere disappeared, whereas those in the ischemic hemisphere steadily increased in number in a narrow rim around the lesion border. Given that a significant proportion (25%) of transplanted NPCs in the mouse brain expressed the proliferation marker Ki67 at 3 days post-transplantation, this selective accumulation may be explained in part by the local proliferation of transplanted cells.

Beyond the proliferation of grafted NPCs, their emergence from peripheral organs (where they may have previously homed), such as the lungs, liver and spleen (Lappalainen et al., 2008), may also contribute to the delayed accumulation of systemically injected cells around the stroke lesion. While the localization of cells of a neural lineage within peripheral organs may carry health risks related to malignant transformation, the idea that NPCs have the capacity to exert their therapeutic efficacy via peripheral actions is intriguing. When fetal human NPCs were delivered intravenously in rats submitted to collagenase-induced intracerebral haemorrhage (ICH), these cells were identified primarily in secondary lymphoid organs where they induced a reduction in the levels of inflammatory mediators and activated macrophage numbers (Lee et al., 2008). This effect was found to be therapeutically relevant as splenectomy, performed before ICH, abolished the effects of NPC transplantation on both brain oedema and inflammatory infiltrates (Lee et al., 2008). This finding, combined with observations that transplanted NPCs can hamper the activation of myeloid dendritic cells (DCs) and restrain the expansion of antigen-specific T cells in inflammatory CNS conditions (Pluchino et al., 2005, 2009), supports the putative ability of systemically delivered NPCs in modulating important aspects of the stroke-induced activation of innate and adaptive immune responses.

It is becoming clear that the therapeutic effect of intravenous cell delivery is independent of the amount of NPCs that is achieved within the brain, while local intracerebral NPC transplantation outcomes strictly depend on the amount of NPCs that reside within the lesion site. Consequently, intracerebral transplantation of NPCs is successful only if grafted NPCs within the ischemic brain survive in adequate quantity. The major determinant of the survival of locally transplanted cells within the ischemic brain is the timing of delivery. In a recent study it has been shown that while NPC proliferation, migration, and neuronal differentiation did not differ when cells were intrastriatally transplanted in the subacute (48 h) or chronic phase (6 weeks) after stroke, NPC survival was strikingly reduced following delayed cell delivery (Darsalia et al., 2011). The reasons underlying these findings are largely unknown. It is known that the majority (approximately 80%) of adult-born neurons arising from the endogenous neurogenic niche after stroke die before integrating in the ischemic tissue (Arvidsson et al., 2002), and that the inflammatory milieu plays a pivotal role in this phenomenon. As such, the treatment with anti-inflammatory agents (e.g., indomethacin or minocycline) that suppress microglial activation result in the preservation of these newly formed neurons (Hoehn et al., 2005; Liu et al., 2007). Similarly, the survival of intrastriatal grafts may be strictly dependent on the local inflammatory milieu and, as such, intraparenchymal transplantation should take place before microglial cells are fully activated.

## FUNCTIONAL INTEGRATION OF TRANSPLANTED NPCs

The route of NPC administration has little effect on the phenotype of cells that accumulate inside the ischemic brain, as transplanted NPCs via local or systemic routes retain a profile of characteristics that is similar to those observed *in vitro* prior to transplantation (Jin et al., 2005). This may either be interpreted as evidence that NPCs are able to retain their character, despite the manipulations brought about by transplantation, or that the ischemic microenvironment arrests the capacity of the transplanted NPCs to differentiate into mature neurons or glia. It has indeed been shown that when neuroinflammation predominates, transplanted cells retain an undifferentiated phenotype as a result of the release of soluble mediators (e.g., noggin) by blood-borne inflammatory cells, activated endothelial cells, and astrocytes (Pluchino et al., 2005; Martino and Pluchino, 2006). It is therefore reasonable to speculate that when inflammatory signals begin to fade, transplanted cells are subsequently enabled to differentiate into post-mitotic CNS cells.

We have shown that at 3 and 10 days post transplantation the majority of intravenously transplanted NPCs exhibit an undifferentiated phenotype, lacking lineage-specific markers such as microtubule-associated protein (MAP)-2, DCX, glial fibrillary protein (GFAP), and the oligodendroglial transcription factor (Olig)-2 (Bacigaluppi et al., 2009). Interestingly at 30 days post transplantation, when inflammation was down regulated, the number of transplanted NPCs expressing Olig2 and DCX increased (albeit to only 4.4 and 0.8%, respectively, of the total), whereas the majority of the transplanted cells still exhibited an undifferentiated morphology in the brain tissue. Understanding the mechanisms which foster graft differentiation and reduce the quantity of NPCs restricted to an undifferentiated state (once their therapeutic bystander effects have been fully exploited) is of pivotal importance for future cell-replacement therapies in stroke.

Valuable insights into the abovementioned may eventually come from the observation of the spontaneous differentiation of endogenous neuroblasts after ischemic stroke (Kernie and Parent, 2010). It has been reported that the majority of neuroblasts after ischemia give rise to striatal medium spiny neurons (Parent et al., 2002). This response can be modulated by the addition of growth factors (such as angiopoietin or EGF), which increase the number of differentiated neurons and/or drive the fating of specific neuronal subtypes (i.e., parvalbumin-expressing interneurons; Teramoto et al., 2003; Liu et al., 2009). The potential of pushing NPCs via growth factors toward specific neuronal subtypes has been exploited by a recent study on induced human pluripotent stem (iPS) cells, which were fated before local transplantation to obtain functional cortical neurons *in vivo* (Tornero et al., 2013). Despite major methodological advances associated with this approach, definitive proof regarding the additional value of cell fating on behavioral recovery, when compared to non-fated cellular grafts, is still lacking (Pluchino and Peruzzotti-Jametti, 2013).

## BYSTANDER EFFECTS OF TRANSPLANTED NPCs

Functional recovery of stroke-induced deficits has similarly been reported after intracerebral, intracerebroventricular, or intravenous NPC delivery (Bliss et al., 2007; Doeppner et al., 2012). As already noted, the common finding to all routes of cell delivery is the undifferentiated state in which the majority of transplanted cells are found within the brain parenchyma (Jin et al., 2005). As a matter of fact, the therapeutic potential of NPCs seems to be initially independent of cell differentiation and rather relies on the multiple bystander mechanisms exerted by adult NPCs, which serve to boost restorative responses in the brain and modulate the injured microenvironment (Martino et al., 2011).

We have shown that adult mouse NPCs reduced inflammatory responses in the ischemic brain, thereby preventing delayed neuronal degeneration and brain atrophy, even when transplanted intravenously as late as 3 days post MCAO (Bacigaluppi et al., 2009). Iba-1^+^/MHC class II^+^ microglial activation was reduced upon transplantation of adult NPCs, as was GFAP^+^ astroglial scar formation in the infarct rim (Bacigaluppi et al., 2009). On the histochemical level, dopamine-2 receptor^+^, and cAMP regulated phosphoprotein (DARPP)-32^+^ medium spiny neurons were protected against delayed degeneration in the striatum, which is particularly sensitive to intraluminal MCAO (Bacigaluppi et al., 2009). Diminished delayed neuronal degeneration was noticed not only inside the ischemic lesion but also at distance to it, as the corpus callosum (CC) of mice receiving transplants of adult mouse NPCs was significantly thicker than those of control mice at 30 days post transplantation (Bacigaluppi et al., 2009).

Further, transplanted NPCs were identified in close contact with von Willebrand factor^+^ endothelial cells, CD45^+^ leucocytes and F4/80^+^ macrophages (Bacigaluppi et al., 2009). On the molecular level, pronounced down-regulation of messenger transcripts of inflammatory signals (*IFN-*γ, *TNF-*α, *IL-1*β), regulators of glial proliferation and reactivity (*bFGF*, *vimentin*) and neuronal death and plasticity (*caspase-3*, *growth associated protein-43*, *versican*) was observed in the brains of ischemic mice receiving intravenous transplantation of adult mouse NPCs while a single transcript was up-regulated by adult mouse NPCs, which was the spiny neuron marker *DARPP-32* (Bacigaluppi et al., 2009). These data clearly suggest that NPCs modulate their microenvironment via inhibition rather than activation of transcriptional processes.

Reduced delayed neuronal degeneration and CC atrophy were also noticed with the grafting of human fetal NPCs into the ipsilesional cortex of rats at 7 days following distal MCAO (Andres et al., 2011b). Dendritic branching, as evaluated by Golgi staining, was enhanced by human fetal NPC transplantation, as was contralesional corticospinal axonal sprouting (Andres et al., 2011b). Accumulation of amyloid precursor protein was reduced by human fetal NPC transplantation, pointing toward the restoration of axonal transport processes (Andres et al., 2011b).

Although both systemic and intracerebral transplantation improve neurological recovery, the restorative effects of each route of transplantation exhibit important differences with regard to the potential to influence injured tissue via bystander effects. The local intracerebral grafting of adult mouse NPCs in the brain parenchyma is associated with elevated brain concentrations of BDNF, FGF, and VEGF in the subacute stroke phase, i.e., at 4 days after MCAO in mice (Doeppner et al., 2012). Notably, such elevated growth factor levels could not be observed in the ischemic brain after systemic intravenous NPC delivery, but were still present for as late as 2 months after intracerebral transplantation of NPCs transduced with heat shock protein (Doeppner et al., 2012).

## CONCLUSION

Considering the lack of therapeutic options that promote brain remodeling and neurological recovery after stroke, there is a clear need to reevaluate therapeutic strategies and treatment modalities within the stroke field. Accumulating evidence suggests that beyond the recanalization of blood vessels (by means of thrombolytic therapies), it will not be possible to promote neurological recovery post-ischemia via the modulation of single targets (Hermann and Chopp, 2012).

NPCs possess unique characteristics that differ drastically from conventional therapies (namely pharmacological small molecule compounds, recombinant growth factors and/or antibody-based therapeutics). Transplanted stem cells can promote tissue regeneration by sensing diverse signals in the brain microenvironment, migrating to specific sites of damage, integrating inputs and executing complex response behaviors all aimed at the remodeling/protection of injured ischemic tissue (Fischbach et al., 2013).

While the local delivery of NPCs is able to achieve high levels of protective mediators (e.g., growth factors) in the ischemic brain tissue, questions remain about the feasibility of such surgically invasive procedures in the clinical setting. One might therefore consider systemic NPC transplantation, which is minimally invasive. In view that systemically administered NPCs do possess potent anti-inflammatory effects, promote brain remodeling and induce functional neurological recovery in rodents, this option could be indeed of clinical value.

Future studies will have to clearly define the safety and efficacy of NPC transplantation after both systemic and local delivery. Such knowledge will increase our understanding of cellular therapies and in turn guide future translational strategies that are urgently needed to promote brain remodeling and repair in stroke patients.

## Conflict of Interest Statement

The authors declare that the research was conducted in the absence of any commercial or financial relationships that could be construed as a potential conflict of interest.

## ABBREVIATIONS

BDNF: brain-derived neurotrophic factor
BMP: bone morphogenetic protein
CCL: CC chemokine ligands
CSPGs: chondroitin sulfate proteoglycans
CNTF: ciliary neurotrophic factor
GDNF: glial cell line-derived neurotrophic factor
HO-1: heme oxygenase 1
IFN-γ: interferon-γ
IGF1: insulin-like growth factor-1
IL: interleukin
LIF: leukemia inhibitory factor
MMPs: matrix metalloproteinases
NGF: nerve growth factor
NO: nitric oxide
NPCs: neural precursor cells
NT-3: neurotrophin-3
PGE: prostaglandin E
SDF-1α: stromal cell-derived factor-1α
SHH: sonic hedgehog homolog
SVZ: subventricular zone
TIMPs: tissue inhibitors of metalloproteinases
TN-C: tenascin-C
TNF-α: tumor necrosis factor-α
TSP: thrombospondin
VEGF: vascular endothelial growth factor
